# Somatic glypican 3 (*GPC3*) mutations in Wilms' tumour

**DOI:** 10.1038/sj.bjc.6600417

**Published:** 2002-06-17

**Authors:** G R M White, A M Kelsey, J M Varley, J M Birch

**Affiliations:** Cancer Research UK Cancer Genetics Group, Paterson Institute for Cancer Research, Wilmslow Road, Manchester M20 4BX, UK; Department of Histopathology, Royal Manchester Children's Hospital, Manchester M27 1HA, UK; Cancer Research UK Paediatric & Familial Cancer Research Group, Royal Manchester Children's Hospital, Manchester M27 4HA, UK

**Keywords:** Wilms' tumour, glypican 3, mutation

## Abstract

Tumour and normal tissue from 41 male cases of Wilms' tumour were screened to determine the presence of sequence variants in the glypican 3 (*GPC3*) gene. Two non-conservative single base changes were present in tumour tissue only. These findings imply a possible role for *GPC3* in Wilms' tumour development.

*British Journal of Cancer* (2002) **86**, 1920–1922. doi:10.1038/sj.bjc.6600417 www.bjcancer.com

© 2002 Cancer Research UK

## 

It has been known for many years that the risk of Wilm' tumour (WT) is greatly increased in children with certain congenital abnormalities and syndromes, but the complexity of WT genetics has only been recognised more recently (Hastie, 1994). Thus far the only WT gene to be fully characterised is *WT1*, located on chromosome 11p13 but deletion or mutation to *WT1* is found in only about 20% of sporadic WT (Huff, 1998). Beckwith-Wiedemann syndrome (BWS) is characterised by pre-and/or post-natal overgrowth and a variety of other congenital abnormalities and confers a greatly increased risk of WT (DeBaun and Tucker, 1998). Other overgrowth syndromes may also predispose to WT. Numerically the most important of these is Simpson-Golabi-Behmel syndrome (SGBS) which shows an X-linked pattern of inheritance. WT has been noted in a number of patients with SGBS (Xuan *et al*, 1994, 1999; Hughes-Benzie *et al*, 1996; Lindsay *et al*, 1997).

Constitutional deletions or mutations in the glypican 3 gene (*GPC3*) at Xq26 are found in many SGBS families (Pilia *et al*, 1996). This suggests that deletion or mutation in *GPC3* may be involved in the development of some WT cases. The possibility that somatic mutations to *GPC3* may be present in sporadic WT not associated with SGBS therefore also arises. To investigate this possibility we have analysed an unselected series of WT cases, for the presence of somatic and constitutional alterations to *GPC3* in tumour and normal tissue respectively.

## MATERIALS AND METHODS

Approval for the project was given by the relevant research ethics committees. Histopathological material from incident cases of WT included in the Manchester Children's Tumour Registry (Blair and Birch, 1994) was examined. Paraffin blocks containing tumour tissue and normal kidney respectively, were selected for each case. To simplify the analysis male cases only were included in the study.

One to two 10-micron sections from each block were placed in individual tubes and DNA extracted as described (Varley *et al*, 1999). Oligonucleotide primers were custom synthesised by Life Technologies to a standard purity. Table 1

**Table 1:**
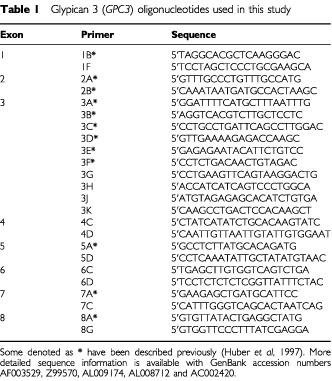
Glypican 3 (*GPC3*) oligonucleotides used in this study

details the main *GPC3* primers used in this study. PCR reactions were carried out using a Techne GeneE Thermal Cycler. A standard protocol of 100 μ1 reactions, containing 0.5 μg of each primer, either 1× *Taq* buffer (Roche) or 1× TNK 100 buffer (Blanchard *et al*, 1993), 100 μM dNTPs and 3 units of *Taq* polymerase, with or without 5% DMSO, was run for one cycle at 94°C for 3 min, followed by 38 cycles of 94°C for 1 min, annealing temperature (see Table 2

**Table 2:**
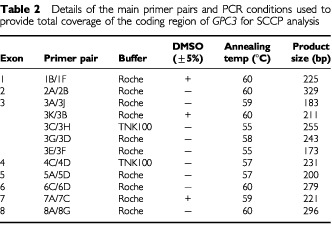
Details of the main primer pairs and PCR conditions used to provide total coverage of the coding region of *GPC3* for SCCP analysis

) for 1 min, and 72°C for 1 min. A final extension at 72°C for 10 min was given at the end of the last cycle. Products from the same PCR reaction could be used for both SSCP analysis and subsequent sequencing.

Table 2 shows the primer pairs used for SSCP, providing total coverage of the coding region of *GPC3*. One μ1 of PCR product was subjected to a second round of amplification in a 10 μ1 reaction (10 cycles at 94°C for 1 min, 50°C for 1 min, and 72°C for 1 min with the final cycle having an extension time of 10 min) as described previously (Varley *et al*, 1999). Initially, tumour material only was examined. Where a variant was identified DNA from the corresponding normal tissue was analysed and the fragment sequenced. Controls (no DNA and normal DNA) were included on every gel. Any samples showing abnormal band shifts were re-analysed by repeating both first- and second-round SSCP–PCR reactions.

PCR products were purified using a Wizard PCR Preps DNA Purification System (Promega). Approximately 100 ng of this DNA was labelled with Big Dye Terminator DNA Sequencing Kit (ABI) using a Perkin Elmer 480 DNA Thermal Cycler and ABI protocols and sequenced using an ABI 377 system. The splice site prediction program made available from the Berkeley *Drosophila* genome project (http://www.fruitfly.org/seq_tools/splice.html) was applied routinely with any sequence variant to address the possibility of a new splice site being created.

## RESULTS

**Figure 1:**
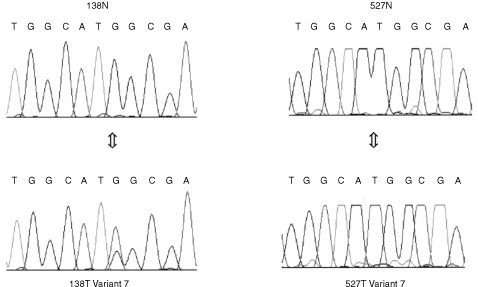
Sequencing electropherograms of variants 6 and 7. Sample 138 shows a C–T transition, not present in the normal, which changes a basic histidine to an uncharged polar tyrosine at position 558. The variant in sample 527 is a G–A transition at position 1902 which changes the non-polar alanine to the polar threonine. Note that sample 138 is shown sequenced on the reverse strand.

Paired tumour and normal tissue samples were available on 41 male cases. Seven different variants were detected. Variant 4 was present in three cases but the remaining six variants were detected in single cases only. Variants 1–5 were present in both tumour derived and the corresponding normal DNA. Variants 6 and 7 were detected in tumour derived material only (Figure 1

).

Variants 1 and 3 were present in the same case. Variant 1 is due to a silent T–C transition in exon 7, position 1697 (all nucleotide numbering refers to the sequence described by Huber *et al*, 1997). Variant 3 is due to a silent A–G transition in exon 8 position 1823. Both variants were reported in three out of 13 ovarian cancer cell lines, and always occur together (Lin *et al*, 1999). Variant 2 is the result of a G–A transition in exon 5, position 1482, which changes non-polar valine to non-polar methionine. We have observed the same sequence variant in blood samples from two out of 36 unselected breast cancer patients (i.e. 72 X chromosomes). Variants 4 and 5 are the result of the reduction in a run of 16 Ts in the splice acceptor region of exon 3–15 and 14 respectively. We consider variants 1–5 to be polymorphisms with no predicted effect on splicing.

Sequencing showed variant 6 to be due to a C–T transition in exon 3 position 558. This changes a basic histidine to an uncharged polar tyrosine. Variant 7 is due to a G–A transition in exon 8, position 1902. This changes the non-polar alanine to the polar threonine. Variants 6 and 7 are tumour specific i.e. they were present in tumour DNA but were not detected in DNA from normal kidney and may represent somatic mutations.

## DISCUSSION

*GPC3* is one of six glypican genes which so far have been identified in vertebrates. Glypicans are highly evolutionarily conserved cell surface proteins which have a role in morphogenesis and growth regulation. Glypican 3, encoded by the *GPC3* gene, is a GPI-linked heparan sulphate proteoglycan. It is highly expressed in embryonic mesodermal tissues corresponding to the tissues showing overgrowth or other abnormalities in SGBS (Selleck, 2000; De Cat and David, 2001). It is the only glypican to date for which mutations in humans have been documented.

We have detected somatic point mutations in the *GPC3* gene in two out of 41 WT cases (4.9%). This represents the first fully documented report of such mutations in tumour tissue and provides evidence that disruption of the GPC3 protein may be involved in initiation, development or progression in some Wilms' tumours. Support for such a role is provided by the observation that GPC3 is expressed in WT tissue but not in the corresponding normal kidney (Saikali *et al*, 2000). The 4.9% GPC3 mutation frequency detected in the present series may be an underestimate since the methods used did not include analysis of the promoter region and would not have detected large deletions of *GPC3* exons which occur in some SGBS patients (Hughes-Benzie *et al*, 1996; Pilia *et al*, 1996; Lindsay *et al*, 1997). Southern blotting is not suitable for the archival material available to us, but we have partially addressed this issue by carrying out multiplex PCR analysis of *GPC3* exons and a control gene for 20 of the WT samples. Results (data not shown) showed no evidence for deletions.

The mutation in exon 8 occurs near to the C terminus in a predicted region of glycosylphosphatidylinositol (GPI) anchorage. A number of deletions detected in patients with SGBS also affect exon 8 and probably prevent attachment of any product to the cell membrane (Hughes-Benzie *et al*, 1996; Pilia *et al*, 1996; Lindsay *et al*, 1997). Furthermore, the GPI-anchoring domain has been identified as critical for the induction of apoptosis in mesothelioma and breast cancer cell lines (Gonzalez *et al*, 1998). The mutation in exon 3 does not appear to be in any known functional domain but is non-conservative and may be expected to affect protein conformation. The five published cases of WT in patients with SGBS in whom alterations in the *GPC3* gene have been detected were all associated with constitutional deletions in exon 1 and/or exon 2 (Hughes-Benzie *et al*, 1996; Pilia *et al*, 1996; Lindsay *et al*, 1997). Additional studies are required to establish the functional consequences of these putative somatic mutations.

In conclusion in this preliminary study, we have identified somatic mutations in the *GPC3* gene in two of 41 cases of WT, providing evidence of a link between developmental genes and embryonal tumours. Further investigations of the possible role of GPC3 in WT are indicated.
